# Accelerometer Based Data Can Provide a Better Estimate of Cumulative Load During Running Compared to GPS Based Parameters

**DOI:** 10.3389/fspor.2020.575596

**Published:** 2020-10-30

**Authors:** Benedicte Vanwanseele, Tim Op De Beéck, Kurt Schütte, Jesse Davis

**Affiliations:** ^1^Human Movement Biomechanics, Department of Movement Sciences, KU Leuven, Leuven, Belgium; ^2^Department of Computer Science, KU Leuven, Leuven, Belgium

**Keywords:** running, wearable, cumulative load, speed, distance

## Abstract

Running is a popular way to become or stay physically active and to maintain and improve one's musculoskeletal load tolerance. Despite the health benefits, running-related injuries affect millions of people every year and have become a substantial public health issue owing to the popularity of running. Running-related injuries occur when the musculoskeletal load exceeds the load tolerance of the human body. Therefore, it is crucial to provide runners with a good estimate of the cumulative loading during their habitual training sessions. In this study, we validated a wearable system to provide an estimate of the external load on the body during running and investigated how much of the cumulative load during a habitual training session is explained by GPS-based spatiotemporal parameters. Ground reaction forces (GRF) as well as 3D accelerations were registered in nine habitual runners while running on an instrumented treadmill at three different speeds (2.22, 3.33, and 4.44 m/s). Linear regression analysis demonstrated that peak vertical acceleration during running explained 80% of the peak vertical GRF. In addition, accelerometer-based as well as GPS-based parameters were registered during 498 habitual running session of 96 runners. Linear regression analysis showed that only 70% of the cumulative load (sum of peak vertical accelerations) was explained by duration, distance, speed, and the number of steps. Using a wearable device offers the ability to provide better estimates of cumulative load during a running program and could potentially serve as a better guide to progress safely through the program.

## Introduction

Recreational distance running is one of the most popular forms of physical activity (Pedisic et al., 2019). Approximately 20% of all people in Western countries go out for a run once or twice a week. By doing so, runners profit from the associated health benefits which range from improvements in mental health to prevention of chronic diseases. Hence, recreational running can be considered as an ideal lifestyle medicine (Lee et al., 2017). However, runners must receive the right running dose to reap these health benefits. Getting the dose right can be tricky as the dose must exceed the minimal amount of running needed to receive its health benefits whereas running too much can lead to running-related injuries (RRIs) (Bertelsen et al., 2017).

Despite the benefits of running, the development of RRIs remains a major problem as the incidence of RRIs remains high with reports of an average of 17.8 injuries per 1,000 h of running (Videbæk et al., 2015) or with 40% of novice runners having a RRI during the first year after a start-to-run program (Kluitenberg et al., 2015). RRIs are generally musculoskeletal overuse injuries at the legs that result from the repetitive motion pattern of running in which the legs experience high amounts of musculoskeletal loading during every step. The etiology of RRIs is multifactorial and a great diversity of intrinsic related (such as age, gender, BMI) and extrinsic (such as training load) risk factors have been identified. However, most studies identifying these factors show inconsistent or conflicting results. Bertelsen et al. (2017) recently proposed a conceptual framework to inform future RRI prevention studies. This framework explains that a RRI occurs when the musculoskeletal load capacity, i.e., the load that can be sustained before a RRI occurs, is exceeded by the cumulative load, i.e., the sum of stride-specific mechanical loads that musculoskeletal structures are exposed to during a running session. They recommend that future RRI studies should quantify participation objectively by reporting the number of strides per training session as well as the structure-specific load per stride. However, they do recognize that structure-specific load per stride is currently very challenging to measure and cannot be performed during each training session of the runner.

As it is crucial to appropriately monitor the cumulative load as a product of load magnitude and load volume, proxies of this load have been used to develop training programs aimed to minimize the risk of overuse injuries by prescribing the right training dose. Most running programs use a 10% increase as a progression rule, where the load progression is typically based on time or distance (Buist et al., 2008; Bredeweg et al., 2012). A recent literature review by Edwards (2018) provided very limited evidence to support that a sudden change in training load is associated with an increased risk of a running-related injury. However, changes in training load were mainly defined as proxies of the volume (such as distance, duration, or number of steps) and did not capture the magnitude of the musculoskeletal load (such as muscle forces or joint contact forces). A recent mechanobiological model of Edwards (2018) showed that mechanical fatigue of the musculoskeletal tissues is dependent on the number of cycles multiplied by the load magnitude to the 5–9th power depending on the tissue involved. As such, the load magnitude will have more of an effect than the volume in the emergence of RRIs. Traditionally, load magnitude during running is determined in highly sophisticated laboratory settings which combine ground reaction force measurements (e.g., force-instrumented treadmills) with motion analysis (e.g., 3D motion capture). Although these give very detailed information on the tissue specific loading, they provide only a “snapshot view” over a limited number of running strides confined to the laboratory environment. Vertical ground reaction forces (vGRF) have also been used to estimate the loading on the musculoskeletal system. Although easier to measure, it is still not possible to measure these in the normal training environment of the runner.

Wearable technology in terms of inertial measurement units (IMU's) is capable of capturing biomechanical data in real-life environments. Using a wearable trunk accelerometer it is possible to obtain ecologically valid estimates of peak vGRFs outside the lab, as previously shown (Neugebauer et al., 2014; Neugebauer and Lafiandra, 2018). However, this was only done during walking (Neugebauer and Lafiandra, 2018) or using accelerometers with a low range (Neugebauer et al., 2014). It is therefore still crucial to estimate the accuracy of the estimates of peak vGRFs during running. Using these estimates of vGRF in combination with number of steps could have the potential to estimate cumulative loading. The potential of such an approach was shown for the first time by Kiernan et al. (2018) in a small cohort study. In this study, the 3 runners who sustained an injury had higher weighted cumulative loading per run measured by a hip mounted accelerometer compared to those who didn't sustain an injury. However, the added value of using cumulative loading compared to the commonly used estimates such as distance, duration, speed, and number of steps is still unknown. Therefore, the goal of the current study is to explore how well distance, duration, speed, and number of steps explain cumulative loading during habitual running sessions. For that, we first aimed to validate the use of vertical acceleration measured using our in-house developed wearable system using an indoor instrumented treadmill, and then to determine how much of the cumulative load during a habitual running session can be predicted using GPS based spatiotemporal parameters.

## Methods

### Indoor Validation

Nine habitual runners were invited to the Movement and posture Analysis Laboratory of Leuven (Belgium) to determine the validity of accelerometer-based parameters to estimate peak vGRF. Prior to the experimental testing, participants had a 5-min warm-up on a motorized force measuring treadmill (Motekforce Link, Amsterdam, The Netherlands). Next, they ran for 1 min at three incremental speeds (2.22, 3.33, and 4.44 m/s) in sequential order with 2-min rest periods between speed intervals. Ground reaction forces during running were measured using the force plate embedded in the treadmill with a sampling frequency of 1,000 Hz. Force plate data was first low-pass filtered in MatLab (Mathworks, Natick, US) using a third order Butterworth filter with a cut-off frequency of 45 Hz to avoid smoothing out high-frequency impact transients then parsed into individual steps. vGRFpeak was defined as the maximum vGRF during 40–60% stance. Foot contact was defined as vGRF exceeding 50 N. Ground reaction forces were normalized to body mass.

In addition, the runners were equipped with a custom-made wearable system. The wearable system consists of a smart phone (Nokia 1) attached in an armband on the upper arm of the runner's preferred side, and an IMU (LPMS-B2 wireless Bluetooth 2 ± 16 g range, sampling at 200 Hz, 12 g weight, LP-RESEARCH, Tokyo, Japan) attached using a tightly fitted waistband centered on the lower back at spinal level of L3–L5 (Schütte et al., 2015). The IMU was used to record tri-axial accelerations for each running speed epoch, respectively. To match each running step, IMU acceleration signals were temporally aligned with vGRF signals. This was achieved by cross-correlating the IMU's vertical acceleration profile with the vertical acceleration profile derived from a retro-reflective marker placed on the posterior side of the IMU. Specifically, the marker's trajectory was recorded in 3D at 200 Hz using a 10 camera Vicon system (Vicon, Oxford, Metrics UK) and was time synchronized with the vGRF. Marker acceleration was obtained after differentiating from marker displacement and velocity. Both displacement and velocity were low-pass filtered using a third order Butterworth filter with a cut-off frequency of 20 Hz prior to differentiating. Peak positive accelerations (g) along three longitudinal axes of the trunk: vertical (ACC_v), anteroposterior (ACC_ap), and mediolateral (ACC_ml), were extracted in the time domain and were defined as the maximum absolute acceleration during stance. Step detection and the contact phase was determined based on previously published algorithms (Benson et al., 2019).

### Outdoor Training Monitoring

To determine the association between accelerometer-based cumulative loading and spatiotemporal characteristics, we monitored the habitual training sessions of regular runners during a period of 3 months using the same wearable system as described in 2.1 Indoor Validation. We recruited 96 runners who ran on a regular basis over the previous 6 months and were between 18 and 60 years of age. Participants were excluded from the study if they had any known history of metabolic, neurological, or cardiovascular disease or had any recent (6 months prior) surgery of the lower limbs or back. Recruitment was done through social media, using electronic flyers distributed to local running groups and clubs.

Prior to the first training session, participants were asked to complete a questionnaire including demographics as well as detailing their training and injury history. Participants were asked to meet at the Movement and Posture Analysis Laboratory of Leuven (Belgium) and received clear instructions on how to attach and properly position the belt. This belt had a special attachment such that the sensor fit tightly and comfortable between the left and right posterior superior iliac spines on the lower back. The positioning of the sensor was checked through an automatic calibration procedure while the runners was standing still. All participants were injury free at the time of testing. Written informed consent was received from all runners prior to participation in accordance with the Declaration of Helsinki. The local ethics committee (Medical Ethics Committee of University Hospital Leuven) approved the study (S62086). During the measurements, participants ran according to their habitual training plan, with the addition of an accelerometer-based wearable device provided by the researchers. Specifically, participants were able to self-select their own training parameters (e.g., pace, duration, distance, and terrain/surface). Participants were asked to track their training sessions with the wearable system during a period of 3 months. All signal processing of acceleration curves was performed using the same algorithms as described for the validation study. Algorithms were implemented in Python. Cumulative load per training session was calculated as the sum of all peak vertical accelerations for all steps. Pace, duration, and distance were extracted from the GPS in the mobile phone (as part of the wearable system).

### Statistical Analysis

All statistical analyses were conducted using SPSS software (SPSS; v26.0, SPSS Inc., New York, USA). Statistical significance was set at *p* < 0.05. All data were checked for normalcy using a Kolmogorov-Smirnov Test in combination with visual inspection of the histograms.

For the indoor validation study, correlations between the accelerometer data and the peak vGRF were calculated using Pearson product-moment correlation coefficients when data were normaly distributed. A linear regression model was developed using ACC_v, ACC_mp, ACC_ap and speed as independent variables.

For the outdoor data, cumulative load per training session was calculated for all runners. For a sub-set of runners with more than two training sessions (*n* = 57) a pooled cumulative load per runner was calculated as the average of the cumulative load per training session of that specific runner. In addition, average and standard deviation ACC_v was calculated per training session. For each runner, a pooled average and pooled standard deviation was calculated as the mean ACC_v and mean of the standard deviation of all training session of that runner.

Linear regression models to predict cumulative loading per training session was developed using all runners data (*n* = 96) and a combinations of duration (minutes), distance (m), average speed (m/s), and number of steps as core hypothesized predictors. To assess the real-world predictive performance of the above linear regression model, we used a leave-one-subject-out-cross-validation approach (De Beéck et al., 2018). Thus, for each runner, we trained a linear regression model based on the data of all other runners. This model is used to predict the cumulative load of each held-aside session of that runner. The absolute error of a prediction is then defined as the absolute value of the difference between the true cumulative load of the session and the predicted cumulative load. To aggregate the absolute errors across all sessions and runners, a two-step aggregation procedure to account for the variable amount of sessions per runner was employed (De Beéck et al., 2018). First, a runner's mean individual absolute error was calculated as the average absolute error over all of that runner's sessions. Second, the global mean absolute error across all runners was calculated as the average of all individual mean absolute errors.

## Results

### Indoor Validation Study

**Figure 1:**
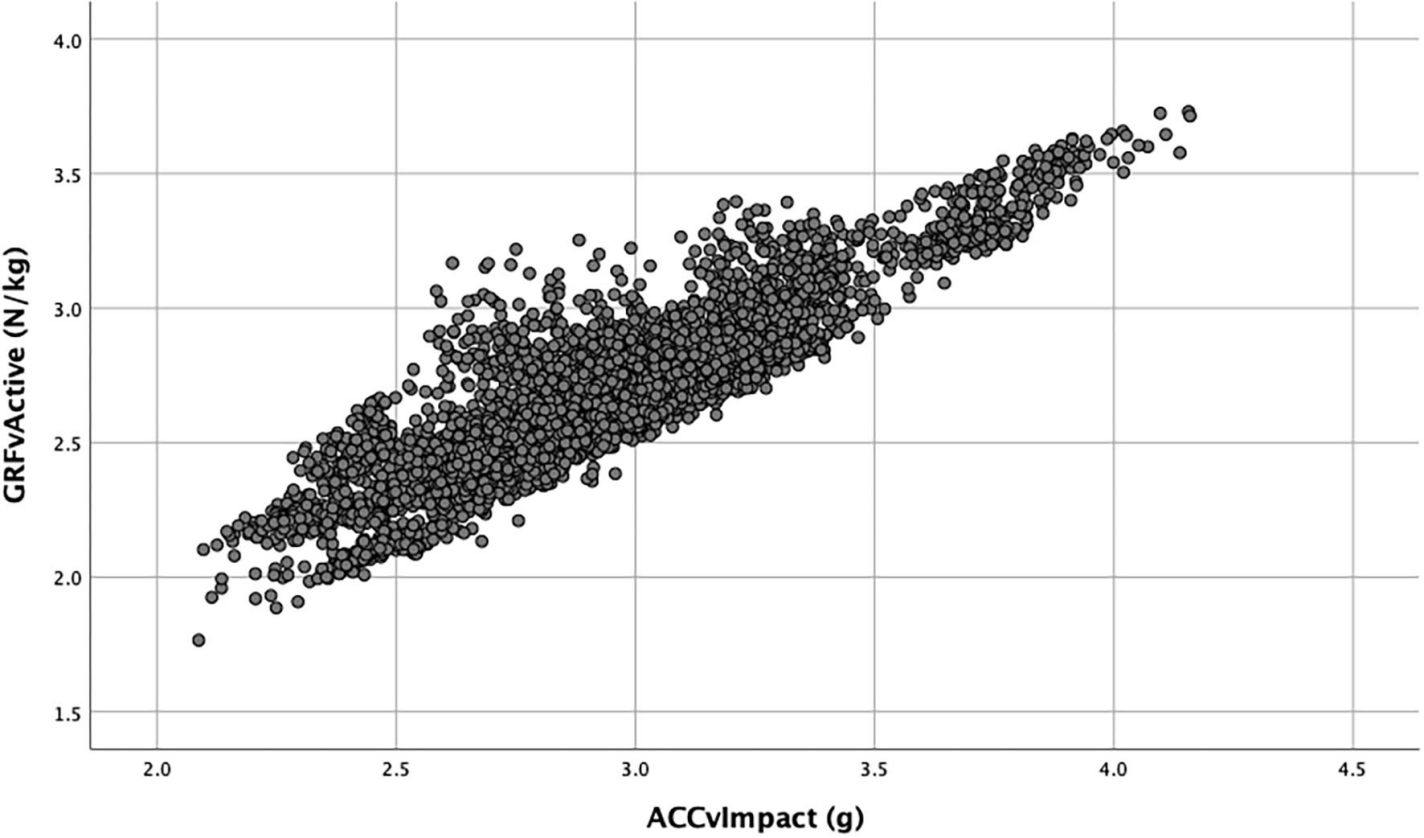
Linear regression between the active peak vertical ground reaction (GRFvActive) and the peak vertical acceleration (ACCvImpact). Each point represents a running step from a runner running on an instrumented treadmill.

To validate the accelerometer data in comparison with the ground reaction force, a total number of 4,158 running steps were used. Average vGRF was significantly correlated with ACC_v, ACC_ml, and ACC_ap (Table 1). ACC_v alone explained 79.7% of the variation in the vGRF_peak (Figure 1).

**Table 1:** Correlations (r∧2) between the peak vertical ground reaction force (vGRF_peak) and the peak acceleration signals in the indoor validation study.

	**2.22 m/s**	**3.33 m/s**	**4.44 m/s**	**R∧2**
vGRF_peak (N/BW)	2.44 ± 0.21	2.71 ± 0.28	2.85 ± 0.31	
ACC_v (g)	2.72 ± 0.27	3.00 ± 0.34	3.03 ± 0.37	0.797
ACC_ap (g)	0.59 ± 0.14	0.78 ± 0.16	0.89 ± 0.19	0.186
ACC_ml (g)	0.33 ± 0.17	0.50 ± 0.26	0.58 ± 0.29	0.064

*Data for the mean ± standard deviation of all parameters during all running trails are reported*.

Linear regression analysis resulted in R∧2 of 0.873. Linear model:

*vGRF_Active* = *0.46* + 0.62^*^ *ACC_v* + *0.81* ^*^ *ACC_ml* + 0.46^*^ACC_ap +*.01* ^*^ *Speed*.

### Outdoor Training Monitoring

Cumulative load was calculated for 96 runners (Table 2) including 498 running sessions with on average 6,640 (2,239–31,870) steps per session. The median duration and distance of a running session was 42 (14–228) min and 7.5 (2.1–37.0) km. Runners ran at an average speed of 3.0 (2.0–4.65) m/s.

**Table 2:** Demographics of the runners included in the outdoor monitoring study.

Gender (male/female)	41:18
Age	29.5 ± 8.1
BMI	22.4 ± 2.2
Number of years running	9.6 ± 7.6
Number of runners with history of RRIs	21

The median (range) cumulative load per session for the sub-group of runners with more than two running sessions was 37,198 (9,518–190,409) g. The median (range) ACC_v per session was 5.42 (2.89–12.99) g for all running sessions. Median standard deviation within one training session was 0.90 (0.34–3.27)g. The median (range) of the pooled average ACC_v per runner was 5.54 (3.17–10.56) g.

The median (range) of the pooled cumulative load per runners was 32,432 (10,332–102,331) g. The median (range) of the pooled standard deviation was 0.52 (0.05–2.79) g.

The R∧2 values between the spatiotemporal parameters and cumulative load per session are represented in Table 3. The best regression model with an r∧2 of 0.703 is represented by

**Table 3:** Overview of the results of the linear regression models with the spatiotemporal parameters to predict the cumulative load during all habitual running sessions.

**Duration**	**Distance**	**Speed**	**Number of steps**	**R∧2**
**(min)**	**(km)**	**(m/s)**		
		x		0.005
	x			0.477
	x	x		0.560
x				0.565
x	x			0.569
x		x		0.571
			x	0.629
		x	x	0.652
	x		x	0.699
x	x		x	0.701
x	x	x	x	0.703

*Cumulative_Loading* = −*1,33* + *13.078* ^*^steps - 7.108 ^*^distance – 13.17 ^*^ *duration* + *1,61* ^*^ *speed*

Using total distance, duration, speed, and number of steps as input for the above linear regression, we found a global mean absolute error of 9,488 g. Compared to an average load of 35,271 g per session (i.e., aggregated using same two-step aggregation), this yielded a relative error of 26%. When only distance, duration, and speed were being used, the global mean absolute error of the models increased to 10,908 g (31%).

## Discussion

The primary aim of this study was to investigate how much of the cumulative loading during a training session is explained by commonly used spatiotemporal characteristics such as speed, distance and duration. We found that only 70% of the cumulative loading per training session was explained by these spatiotemporal parameters and that using a generic program based on these parameters will result in a relative error of 26%.

Monitoring load during training sessions gained significant attention over the last decade especially as too high training loads and rapid changes in training loads have been associated with injury. Using training load researchers, coaches, and trainers attempt to quantify the external load on the human body during physical activity (Foster et al., 2017). Most studies represented training load, especially in running, by the duration, distance run, or number of steps (Buist et al., 2008; Nielsen et al., 2013, 2014). However, based on our results these spatiotemporal parameters only provide a very rough estimate of this load (<70%). Other factors besides spatiotemporal parameters such as running surface (Schütte et al., 2016), fatigue (Dierks et al., 2010; Schütte et al., 2015), or other contextual factors have previously been shown to have an effect on the external load on the human body.

The availability of wearable sensors enables obtaining more detailed information about the external load in the ecological environment of the runner during each running session. Although the axis system of the sensors could have been slightly different from the world coordinate system used for the GRF, the current study demonstrates that using peak accelerations during the contact phase of running provides a good estimate of the peak vGRF. For the indoor validation study, the same analysis was performed except that tilt correction was included. This yielded very similar results (data not shown). Because omitting tilt correction yields a simpler and more computationally efficient implementation of the algorithms, therefore results without tilt correction were reported. Our results are similar to previous research associating peak vertical acceleration with vGRF during walking (Neugebauer et al., 2014; Neugebauer and Lafiandra, 2018). Predictions of vGRF were accurate to within 4% based on accelerometer data during walking at different speeds while carrying different loads (Neugebauer and Lafiandra, 2018). In contrast, Rowlands et al. (2004) found no significant correlation between peak acceleration and peak impact force. However, they investigated walking and running at different speeds as well as several jumps using a commercially available accelerometer with a low range on the hip. They described in their discussion that the acceleration signal frequently reached the maximum range of 6 g. This studied used an accelerometer with a high range (16 g) and a custom designed sensor attachment, which ensured that the accelerometer only moved with the body. To the best of our knowledge, this is the first study to demonstrate the accuracy of predicting peak vGRF using a single accelerometer securely attached at the lower back. As the vGRF is a good proxy of the external load on the human body, we suggest that training load would be better represented by the sum of the impact loads (measured with accelerometry) of all steps.

In this study, we defined cumulative load as the sum of all peak vertical acceleration of all running steps measured during the habitual training session of 96 runners. This definition is similar to Kiernan et al. although they weighted peak acceleration to the 9th power (Kiernan et al., 2018). Rowland et al. used also peak acceleration to quantify mechanical load (Rowlandsa and Stiles, 2012). The training data showed quite a large variation between runners. For example, the ACC_v was above 10 g for three participants. After carefully checking the quality of the data, it was noticed that all of the recorded session of these runners showed these high values for ACC_v. This might indicate that these high ACC_v are related to the specific running styles of these participants rather than bad data quality due to malpositioning of the sensor. As the participants put the sensor on themselves as instructed in the first session there is a possibility that this affects the data quality. However, the design of the belt as well as the automatic calibration assisted this process and when comparing the data of the first session with the consecutive sessions no difference were observed. Further research is needed to explore if is indeed the case and if this puts these runners at a higher risk of injury. Only 70% of the cumulative loading could be explained by the commonly used spatiotemporal parameters duration, distance, speed, and number of steps. This shows that using accelerometer-based wearable technology gives better estimates of cumulative load which might improve the value of these parameters when designing and modifying training programs. A recent literature review (Damsted et al., 2018) concluded that there is very limited evidence supporting that a sudden change in training load is associated with increased risk of running-related injury. However, changes in training load were defined as changes in speed, distance, volume, or frequency. In contrast, Kiernan et al. (2018) recently showed that using hip-mounted accelerometer to monitor collegiate athletes throughout their entire run, injured runners had higher weighted cumulative loading per run. This shows the potential to use these loading profiles to investigate further the link between training load and running related injuries. To gain further insight monitoring load using this wearable technology is therefore crucial and could be used to adjust training programs.

Although using our wearable system gives more information about the loading compared to the GPS based parameters, it still only uses a proxy of the whole-body rather than the structure-specific musculoskeletal loading. Future research therefore will need to determine the accuracy of the wearable system in estimating GRF is sensitive enough to detect factors such as cumulative loading or changes in loading to detect or predict a higher risk of injury. In addition, as the model of Edwards (Edwards, 2018) describes, the structure-specific loading component is very important and the association between this structure-specific loading and the whole-body loading is not clear yet. However, in order to measure proxies of structure-specific loading, it is expected that multiple sensors or a combination with lab-based measurements are needed. Future research is needed to further develop specific proxies of structure-specific loading. At the moment, monitoring cumulative load using the purposed wearable system has the potential to give first insights in the influence of training load on the development of running related injuries.

## Conclusion

Trunk based accelerometers could give a more accurate estimation of accumulated training load compared to more commonly used parameters such as speed, time, and duration. Using this wearable technology can increase insight in the role of change and progression of training load in the development of RRIs.

## Data Availability Statement

The datasets generated for this study are available on request to the corresponding author.

## Ethics Statement

The studies involving human participants were reviewed and approved by University Hospital Leuven ethics committee. The patients/participants provided their written informed consent to participate in this study.

## Author Contributions

BV, KS, TO, and JD conceived, designed, and coordinated the study. KS and TO collected the data. BV, KS, and TO analyzed the data. BV initially drafted the manuscript. KS, TO, and JD provided useful suggestions in the preparation of the final manuscript. All authors contributed to the article and approved the submitted version.

## Conflict of Interest

The authors declare that the research was conducted in the absence of any commercial or financial relationships that could be construed as a potential conflict of interest.
